# Neuroinflammation and noradrenergic modulation with β2-adrenoceptors: Emerging therapeutic targets for Parkinson’s diseases

**DOI:** 10.4103/NRR.NRR-D-25-00903

**Published:** 2026-01-27

**Authors:** Maria Micaelle Gomes Tavares, Milena Caroline Nunes Monteiro de Carvalho, Mylaine Santos Mendonça, Iasmin de Carvalho Dantas, Katty Anne Amador de Lucena Medeiros, José Ronaldo dos Santos, Auderlan Mendonça de Gois

**Affiliations:** 1 Behavioral and Evolutionary Neurobiology Laboratory, Department of Biosciences, Federal University of Sergipe, Itabaiana, SE, Brazil; 2 Department of Physiology, Federal University of Sergipe, São Cristóvão, SE, Brazil; 3 Nursing Department, Federal University of Sergipe, Lagarto, SE, Brazil

**Keywords:** β-adrenergic, catecholamines, microglial cells, neurodegenerative disease, neuroprotection

## Abstract

Neurodegenerative disorders, such as Parkinson’s disease, are strongly influenced by neuroinflammatory processes and dysfunction of the locus coeruleus-noradrenergic system. The locus coeruleus-noradrenergic system plays a pivotal role in modulating neuroinflammation and maintaining homeostatic regulation in the central nervous system. This review discusses the structural and functional aspects of the locus coeruleus-noradrenergic system and its interaction with immune responses. We examine how neuroinflammation contributes to disease progression, with a focus on glial activation and peripheral-central immune communication. Additionally, we analyze the impact of β-adrenoceptor-targeting drugs, highlighting the contrasting roles of β-blockers and β2-adrenoceptor agonists in neurodegeneration. While β-blocker, particularly non-selective agents like propranolol, have been associated with exacerbated neuroinflammation and Parkinson’s disease risk, β2-adrenoceptor agonists demonstrate neuroprotective effects by modulating microglial phenotypes, reducing α-synuclein aggregation, and enhancing neurotrophic support. Finally, we explore the canonical and non-canonical β2-adrenoceptor signaling pathways implicated in neuroprotection. Collectively, this review supports β2-adrenoceptors as promising therapeutic targets and underscores the need for further studies to elucidate their mechanistic roles in modulating neurodegenerative processes.

## Introduction

Neurodegenerative diseases are among the most common causes of mortality, cognitive impairment, and motor deficits in the elderly worldwide due to progressive loss of neurons in specific regions of the central nervous system (CNS) (Erkkinen et al., 2018; Licher et al., 2019). With the accelerated aging of the global population, there has been a significant increase in the prevalence of neurodegenerative diseases, particularly Alzheimer’s disease and Parkinson’s disease (PD), making aging the primary risk factor for these conditions (Ding, et al., 2022; Ji et al., 2024; Luo et al., 2024; Zhu et al., 2024).

PD is a chronic disorder of unknown defined etiology, characterized by the progressive loss of dopaminergic neurons and dopamine (DA) depletion in the nigrostriatal pathway, causing motor symptoms such as bradykinesia, muscle rigidity, resting tremor, and freezing of gait (Kalia and Lang, 2015; Caminiti et al., 2017; Andica et al., 2018; Miroshnichenko et al., 2018). Historically, the loss of dopaminergic neurons has been associated with intracellular aggregation of alpha-synuclein (α-syn), which is responsible for the formation of Lewy bodies (Braak et al., 2003; Klos et al., 2005; Hawkes et al., 2007; Stefanis, 2012). However, it is now well established that α-syn accumulation may occur in other regions, such as peripheral tissue regions and the locus coeruleus (LC), before affecting dopaminergic neurons (Braak et al., 2003; Hawkes et al., 2007; Borghammer et al., 2022; Dorsey et al., 2024; Andersen et al., 2025).

Dysfunction of the locus coeruleus-noradrenergic system (LC-NE) in the early stages of PD affects peripheral and central homeostatic mechanisms, such as the modulation of immune system, glial cells and other neurotransmitter systems, which appear to accelerate disease progression and the death of dopaminergic neurons (Rommelfanger et al., 2007; Arora et al., 2021; Lin et al., 2024; Mehran et al., 2024; Zhou and Chu, 2024). Additionally, LC-NE system plays a critical role in the modulation of inflammatory factors associated with neurodegenerative diseases, in which deficits in noradrenergic neurotransmission appear to enhance the synthesis of pro-inflammatory cytokines (Alsaady et al., 2019; Laing et al., 2020; Dahl et al., 2023; Le et al., 2025).

Recently, several studies have reported that populations who make chronic use of β2-adrenoceptors (β2-ARs) agonists for the treatment of asthma or chronic obstructive pulmonary disease have a lower risk of developing PD (Mittal et al., 2017; Gronich et al., 2018; Cepeda et al., 2019; Hopfner et al., 2019, 2020; Germay et al., 2020; Tuominen et al., 2023; Williams-Gray et al., 2025). Additionally, experimental studies show that β2-AR modulation is associated with neuroprotective mechanisms in PD (Oei et al., 2010; Mittal et al., 2017; Zong et al., 2019; O’Neill et al., 2020; Khidr et al., 2023; Torrente et al., 2023; de Gois et al., 2025). Interestingly, neurodegenerative diseases are marked by early LC-NE dysfunction and pronounced neuroinflammation throughout disease progression (Evans et al., 2020; Freire et al., 2022; Hutten et al., 2022; Patterson et al., 2022).

In this review, we highlight the role of the LC-NE system in PD progression and compile evidence suggesting that β2-AR modulation mediates neuroprotective mechanisms associated with LC-NE. We also examine the role of β2-ARs in modulating neuroinflammation and its main associated signaling pathway.

## Search Strategy

The literature search for this review was performed until June 2025. We searched the following databases: PubMed, Scopus, Web of Science, and Google Scholar, coupled with an examination of citations from relevant articles. The following keywords and Boolean operators were used in different combinations: “Locus coeruleus” OR “noradrenergic system” OR “norepinephrine” AND “neuroinflammation” AND (“Parkinson’s disease AND neurodegenerative disease”) AND (“β-adrenergic receptors” OR “β1-adrenoceptors” OR “β2-adrenoceptors” OR “β-Blockers” OR “β2-agonist”) AND (“α-adrenergic receptors” OR “α1-adrenoceptors” OR “α2-adrenoceptors”). The search was restricted to English-language articles. Inclusion criteria comprised: (i) original articles or reviews addressing adrenergic modulation of neuroinflammation; (ii) studies in animal models, cellular models, or clinical/epidemiological contexts related to PD; and (iii) articles reporting molecular pathways or therapeutic outcomes of β-adrenergic modulation. Exclusion criteria were: (i) studies unrelated to neurodegenerative mechanisms; (ii) case reports without mechanistic data; and (iii) conference abstracts without peer-reviewed full text. The title and abstract of the studies were analyzed separately by three authors, who excluded articles unrelated to the topic. Moreover, the selected studies were further revised through full-text screening. The final reference list was generated based on relevance to the topics covered in this review.

## Noradrenergic System: Structure and Function

Norepinephrine (NE) is an important neurotransmitter involved in several homeostatic functions of the organism and behavior (Bari et al., 2020; Van Egroo et al., 2022; Hu et al., 2025). NE is produced by neurons located in the LC and released into various brain regions, regulating multiple responses. The LC is the primary and largest nucleus of the noradrenergic system, from which neurons project to various cortical and subcortical areas, as well as to the cerebellum and spinal cord (Tanguay et al., 2023). It is also modulated by afferent from different brainstem and spinal nuclei, with the main inputs originating from the paragigantocellular nucleus and the prepositus hypoglossi nucleus (Aston-Jones et al., 1986; Poe et al., 2020; Slater et al., 2022).

Anatomically, the LC is located bilaterally in the brainstem, lateral to the wall of the fourth ventricle, in the posterior portion of the pons (Swanson, 1976). It appears as a small bluish ban, a tone resulting from neuromelanin expressed by the dense population of noradrenergic neurons that compose this nucleus. Among these neurons, the most prominent morphological types include large multipolar neurons with extensive arborization in the ventral region and thin fusiform neurons in the dorsal region (Schwarz and Luo, 2015; Chandler, 2016; Plummer et al., 2017).

In general, through its projections to various brain regions, the LC-NE system plays a critical role in the regulation of homeostasis and various behaviors, such as attention, memory, learning, sleep/wakefulness, stress, anxiety and emotions (Price et al., 1996; Morris et al., 2020; Breton-Provencher et al., 2021; Gong et al., 2021; Perez-Tejada et al., 2021; Ross et al., 2021; Chen et al., 2023; Hu et al., 2025), through the modulation of α- and β-adrenergic receptors, which are composed of nine G protein-coupled receptor subtypes: α1A, α1B, α1D (coupled to Gq protein), α2A, α2B, α2C (coupled to Gi protein) and β1, β2 and β3 (coupled to Gs protein) (Kurose, 2004; Ippolito and Benovic, 2020). Thus, LC-NE dysregulation can lead to various cognitive and emotional disorders (Berridge and Spencer, 2016; Perez-Tejada et al., 2021).

Although NE is primarily associated with non-motor functions, analyses show that LC-NE also influences motor control. An increased firing rate of LC neurons precedes voluntary movements in motivational situations (Varazzani et al., 2015), and optogenetic stimulation of the LC enhances motor performance and induces plasticity in the motor cortex (Tseng et al., 2024). Additionally, high levels of NE increase the excitability of the motor cortex and spinal motoneurons (Fung et al., 1991; Thorstensen et al., 2024), demonstrating that LC-NE signaling is critical for postural correction and motor responses controlled by descending pathways (Witts et al., 2023).

Thus, LC-NE dysfunction can impair motor control, in addition to causing non-motor symptoms and cognitive decline in neurodegenerative disorders, such as PD, as it affects noradrenergic neurotransmission in different brain regions (Rommelfanger et al., 2007; Lin et al., 2024; Zhou and Chu, 2024).

## Noradrenergic Modulation as a Therapeutic Target in Parkinson’s Disease

Neuronal degeneration, commonly observed in neurodegenerative diseases, is usually caused by several factors that may occur simultaneously, hindering the identification of the initial neurotoxic mechanisms. PD is a multifactorial disease, characterized by a prodromal phase and a motor phase, and currently has no cure. The asymptomatic phase of PD, which occurs many years before the first motor symptoms, combined with the heterogeneity of neurotoxic mechanisms, makes early diagnosis and therapeutic intervention aimed at delaying disease progression particularly challenging (Poewe et al., 2017; Constantin et al., 2023).

PD is primarily considered a dopaminergic disease, and first-line pharmacological treatments focus on restoring DA levels to alleviate motor symptoms caused by striatal DA depletion. However, PD is a multifactorial condition involving dysfunction in multiple neuronal circuits and neurotransmitter systems (Caligiore et al., 2019). As a result, current drugs can improve motor symptoms without halting neurodegeneration or disease progression.

Among the pharmacological treatments recommended in PD, we highlight dopaminergic agonists, with l-3,4-dihydroxyphenylalanine (L-Dopa) being the primary one, combined with dopamine decarboxylase inhibitors (Stowe et al., 2008; Connolly and Lang, 2014; Elsworth, 2020). Also commonly used are monoamine oxidase-B inhibitors and catechol-O-methyltransferase inhibitors (Kalia and Lang, 2015; Leão et al., 2015; Tan et al., 2022), as well as other agents such as amantadine (Hunter et al., 1970), benzodiazepines, and antidepressants, including sertraline and fluoxetine, the latter being primarily prescribed for the treatment of non-motor symptoms (Church, 2021). Although dopaminergic agonists show efficacy in relieving motor symptoms, they do not prevent disease progression and may cause late-onset dyskinesia in some patients, particularly with prolonged L-Dopa use (Vijayakumar and Jankovic, 2016), highlighting the need for more effective therapeutic strategies.

The multifactorial complexity of PD leads to late diagnosis and pharmacological treatment primarily based on dopaminergic agonists, which do not halt disease progression. This underscores the need for new therapeutic strategies that target other neurotransmitter systems also affected by disease, such as the LC-NE system (Braak et al., 2003; Hawkes et al., 2007; Andersen et al., 2025).

The importance of the LC-NE system in CNS homeostatic regulation and behavioral responses is well established (Berridge and Waterhouse, 2003; Bari et al., 2020; Egroo, Van et al., 2022; Hu et al., 2025), however, its role in the progression of neurodegenerative diseases remains unclear. Still, pharmacological modulation of ARs suggests that the LC-NE system may play a significant neuroprotective role.

Norepinephrine is a monoaminergic neurotransmitter belonging to the catecholamine family, synthesized through the conversion of DA to NE by the enzyme dopamine β-hydroxylase (DβH) (Amaral and Sinnamon, 1977). Once released into the CNS, NE acts on α- and β-ARs expressed on presynaptic and postsynaptic neurons. It can then be metabolized by the enzymes monoamine oxidase and catechol-O-methyltransferase or be reuptake into the intracellular environment by the norepinephrine transporter expressed in the presynaptic terminal (Hu et al., 2024). Therefore, understanding the mechanisms involved in NE metabolism, as well as the dynamics of its receptors and transporters, provides a foundation for the development of novel therapeutic strategies for various diseases.

Studies show that elevated levels of NE released by presynaptic terminals in the CNS, either α2-AR blockade or inhibition of NE reuptake, can protect dopaminergic neurons, reduction of microglial activation and pro-inflammatory factors attenuating both motor and non-motor symptoms in an experimental model of parkinsonism (Yssel et al., 2018; Kreiner et al., 2019). Conversely, α2-AR agonists have been shown to exacerbate bradykinesia in patients with PD (Jahanshahi and Rothwell, 2017; Criaud et al., 2022). These findings are further supported by the observation of reduced neuron density in the LC and decreased expression of α2-AR in both motor and non-motor regions of PD patients (Laurencin et al., 2024), suggesting that the preservation of the LC-NE or the pharmacological modulation of its receptors may be essential for the regulation of neuroprotective mechanisms, including neuroinflammation (O′neill and Harkin, 2018; Butkovich et al., 2020; Jovanovic et al., 2022; Dahl et al., 2023; Evans et al., 2024).

Interestingly, administration of the α2-AR agonist guanfacine in the 6-hydroxydopamine-induced parkinsonism model improved non-nociceptive motor functions, reducing hyperalgesia and glial activation (Gao et al., 2024). In aged non-human primates, guanfacine also enhanced attentional performance and working memory (Decamp et al., 2011). Additionally, administration of another α2-AR agonist, dexmedetomidine, restored dopaminergic levels, reduced inflammatory markers, and improved motor deficits in 1-methyl-4-phenyl-1,2,3,6-tetrahydropyridine-induced parkinsonism model (Zhang et al., 2021). These findings suggest that noradrenergic modulation in the CNS may be more complex than previously thought.

Additionally, the norepinephrine transporter, a transmembrane protein present in both the central and peripheral nervous systems, is responsible for the reuptake of extracellular monoamines into the presynaptic neuron, with the highest affinity for NE (Mandela and Ordway, 2006). The norepinephrine transporter has become an important target for pharmacological therapies, as its inhibition increases NE availability and prolongs its action at ARs. This strategy has been employed in the treatment of various disorders, including attention deficit hyperactivity disorder (Briars and Todd, 2016; Ruppert et al., 2022), chronic pain and fibromyalgia (Lunn et al., 2014), as well as anxiety and depression (Rui et al., 2020).

Depression and anxiety, although classified as psychiatric and mood disorders, are also observed in the prodromal phase of PD and are considered part of its non-motor symptoms (Rui et al., 2020). Depressive symptoms often precede the onset of motor signs and are present in approximately 20%–30% of patients (Schrag et al., 2002; GBD 2019 Stroke Collaborators, 2021; Maier et al., 2021), whereas anxiety affects around 52% of individuals with PD, particularly younger patients (Broen et al., 2016).

Depression in PD, as well as other non-motor symptoms such as pain and cognitive impairment (Schapira et al., 2017; Bustelli et al., 2024), may be associated with the early and extensive loss of monoaminergic neurons in the LC (Torrente et al., 2023; Bustelli et al., 2024) and raphe nuclei (Nobis et al., 2023), preceding the degeneration of the dopaminergic system (Braak et al., 2003; Hawkes et al., 2007; Bustelli et al., 2024; Andersen et al., 2025). Consequently, norepinephrine reuptake inhibitors are often prescribed as adjuvants therapy for PD patients to alleviate these symptoms (Dionisie et al., 2021).

Atomoxetine, a selective norepinephrine reuptake inhibitor widely used for the treatment of mood disorders and attention deficit hyperactivity disorder (Elliott et al., 2020), has also been prescribed for patients with PD, as it increases prefrontal cortex activity and reduces impulsivity, thereby improving behavioral performance (Kehagia et al., 2014). Additionally, atomoxetine has been associated with the restoration of brain network organization and modulation of resting-state functional connectivity (Borchert et al., 2019), as well as with reduced freezing of gait in PD (Ono et al., 2016), a behavior strongly influenced by noradrenergic pathway (Factor et al., 2025). A preclinical study also shows that atomoxetine promotes dopaminergic neuroprotection through inhibition of microglia activation and reduction of pro-inflammatory factors (Yssel et al., 2018).

Studies with norepinephrine reuptake inhibitors, such as atomoxetine and reboxetine, have shown improvements in vocal deficits, communication, and anxiety in patients with PD. However, these studies have some limitations, such as evaluating only the acute effect of the drugs, highlighting the need for further research to better understand the underlying mechanisms (Stepp, 2013; Broen et al., 2016; Hoffmeister et al., 2022). Additionally, a study using experimental models of parkinsonism has demonstrated that reboxetine improves depressive and anxiogenic symptoms (Bonito-Oliva et al., 2014), while other serotonin-norepinephrine reuptake inhibitors, such as duloxetine, alleviate non-motor symptoms and improve gait, although they are less effective for treating apathy (Takahashi et al., 2019).

Although these drugs are indicated as adjuvant treatments to alleviate the non-motor symptoms of PD, none of them can prevent neuronal degeneration. Thus, there is a continued search for new therapies capable of targeting the neuropathological mechanisms underlying neuronal death in neurodegenerative diseases.

## Noradrenergic Regulation of Neuroinflammatory Processes

### Noradrenergic-immune interactions in the onset of neuroinflammation

The expression of ARs in various cells types at both central and peripheral level, including neurons, microglia, astrocytes, immune cells, muscle cells, adipocytes, epithelial cells, among others, demonstrates the importance of the LC-NE system in the maintenance of physiological homeostasis, behavioral regulation, and the modulation of neuroinflammation (Scanzano and Cosentino, 2015; Freire et al., 2022; Patterson et al., 2022). Typically, neuroinflammation is triggered by cellular injury or CNS infections, which suppress adrenergic signaling in glial and immune cells, leading to the release of pro-inflammatory cytokines such as tumor necrosis factor-alpha (TNF-α), interferon-gamma, and interleukin-1 beta (IL-1β) (Alsaady et al., 2019; Laing et al., 2020).

The inflammatory dynamics modulated by NE in immune cells reinforce the bidirectional interaction between the brain and the periphery, as peripheral cytokines can affect central mechanisms (Ide et al., 2018; Logsdon et al., 2018; Agirman et al., 2021; Arora et al., 2021; Mehran et al., 2024). Inflammation is generally associated with the phenotype of immune cells, as reviewed by Sharma and Farrar (2020). The immune cells phenotype can be regulated by the activation of β2-ARs (Scanzano and Cosentino, 2015; Ağaç et al., 2018), such that stimulation of these receptors in macrophages can suppress the pro-inflammatory phenotype (M1) and promote an anti-inflammatory phenotype (M2), mediated by phosphoinositide 3-kinase (PI3K), increased secretion of interleukin-10 (IL-10), and enhanced phagocytic activity (Grailer et al., 2014; Ağaç et al., 2018), as well as by reducing NLRP3 (NOD-, LRR- and pyrin domain-containing protein 3) inflammasome activation and IL-1β secretion in central neurons (Freire et al., 2022).

**Figure 1:**
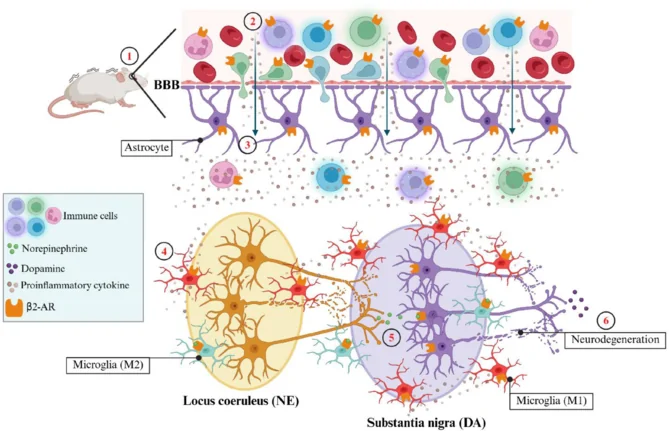
Peripheral–central crosstalk in initiation of neuroinflammation. In neurodegenerative diseases, the disrupted interaction between the noradrenergic system and immune system contributes to the onset of neuroinflammation and progression of neurodegeneration. Immune system dysfunction, which may occur during the prodromal stage of Parkinson’s disease and be caused by various factors, can activate immune cell populations and increase the production of pro-inflammatory cytokines (1). This peripheral dysfunction impairs the blood–brain barrier (BBB), increasing its permeability and allowing the migration of immune cells and pro-inflammatory cytokines into the brain (2 and 3). In the brain, the chronic peripheral pro-inflammatory influence, combined with the pro-inflammatory (M1) phenotype of glial cells, exacerbates degeneration of the noradrenergic system (4), reducing the release of NE, which normally promotes an anti-inflammatory (M2) phenotype (5), thereby accelerating dopaminergic neurodegeneration and DA depletion (6). Created with BioRender.com. β2-AR: β2-Adrenoceptors.

In CD4^+^ and CD8^+^ T lymphocytes and antigen-presenting cells, β2-AR stimulation reduces the release of TNF-α and interferon-gamma (Grailer et al., 2014; Zalli et al., 2015; Sharma and Farrar, 2020) and enhances the proliferation and cytotoxic function of natural killer cells (Diaz-Salazar et al., 2020). Additionally, β2-AR signaling suppresses eosinophil activation and superoxide anion production and modulates oxidative stress responses in neutrophils (Noguchi et al., 2015; Ueda et al., 2020; Berntsen et al., 2022). Therefore, peripheral inflammation is believed to initiate or exacerbate central neuroinflammation through humoral signaling of inflammatory mediators, infiltration of immune cells, and blood–brain barrier (BBB) dysfunction (Golomb et al., 2020; Agirman et al., 2021; Huang et al., 2021; Takata et al., 2021; Mekhora et al., 2024; **Figure 1**).

In this context, due to the strong peripheral and central modulation of inflammation by NE, the LC-NE system emerges as a key link in understanding the mechanisms of neuroinflammation (Butkovich et al., 2018). Evidence indicates that protozoan infections reduce NE and DβH gene expression in various brain regions (Alsaady et al., 2019), reinforcing that the inflammatory response interferes with the neurochemical activity of central neurons. Conversely, modulation of β2-AR reduces the type 2 inflammatory response by negatively regulating the proliferation of innate lymphoid cells induced by helminth infection (Moriyama et al., 2018), suggesting that the interaction between the adrenergic nervous system and the immune system can control multiple inflammatory mechanisms.

Selective activation of the LC-NE system may inhibit neuroinflammation even in the influence of peripheral and CNS inflammation. The expression of β-ARs in central neurons and glial cells allows the LC-NE system stimulation to attenuate the deleterious effects of neuroinflammation (Li et al., 2022) and oxidative stress (Sugama et al., 2019). A series of studies have shown that β2-AR agonists reduce neuroinflammation (Zong et al., 2019; Evans et al., 2020; O’Neill et al., 2020a; Khidr et al., 2023; Torrente et al., 2023), enhance the expression of brain-derived neurotrophic factor (BDNF) and nerve growth factor, and reduce caspase-3 activity (Gleeson et al., 2010).

**Figure 2:**
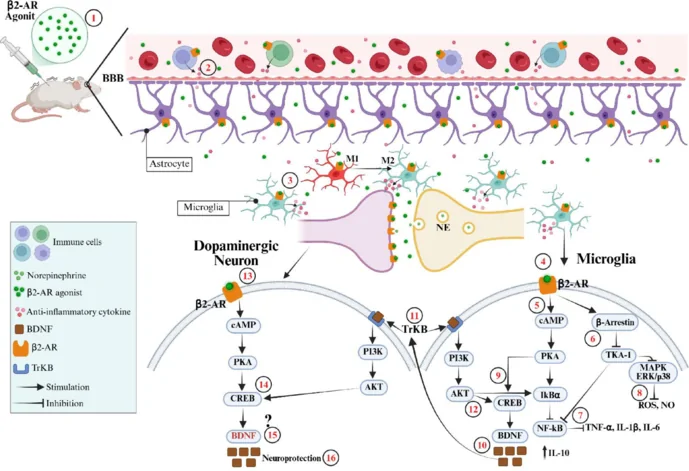
Intracellular signaling pathways mediating neuroprotection via β2-adrenoceptors. Modulation of β2-ARs by norepinephrine or exogenous agonists (1) reduces neuroinflammation by promoting an anti-inflammatory (M2) phenotype in peripherally in the immune cells (2) and glial cells in the brain (3), increasing the production of anti-inflammatory cytokines. Activation of β2-ARs in microglia (4) triggers the canonical signaling cascade (5), leading to increased synthesis of IκBα, via cAMP and PKA, thereby inhibiting NF-κB phosphorylation and reduction of pro-inflammatory cytokine (7). Moreover, through non-canonical pathways (6), β2-AR stimulation can also inhibit pro-inflammatory mediator production (7 and 8) by blocking MAPK signaling via β-arrestin recruitment. Additionally, phosphorylation of CREB (9) via the canonical pathway promotes the expression of BDNF (10), whose synthesis is further enhanced by activation of TrKB (11), via PI3K/AKT/CREB signaling (12). In neuron, stimulation of TrKB (11) and β2-ARs (13) activates signaling cascades that promote neuroprotection by increasing CREB phosphorylation (14), upregulating BDNF expression (15), and supporting neuronal survival (16). Created with BioRender.com. β2-ARs: β2-Adrenoceptors; AKT: protein kinase B; BDNF: brain-derived neurotrophic factor; cAMP: cyclic adenosine monophosphate; CREB: cAMP response element-binding protein; IκBα: inhibitor of nuclear factor kappa B alpha; NF-κB: nuclear factor kappa B; PI3K: phosphoinositide 3-kinase; PKA: protein kinase A; TrKB: tropomyosin receptor kinase B.

The anti-inflammatory effect promoted by β2-AR signaling in the CNS is believed to be associated with inhibition of nuclear factor kappa B (NF-κB), thereby decreasing the synthesis of pro-inflammatory cytokines such as TNF-α (Ryan et al., 2013). Additionally, β2-ARs can act synergistically with Toll-like receptors to stimulate the secretion of IL-10 and reduce pro-inflammatory cytokines such as TNF-α, interleukin-6, and interleukin-12 (IL-6 and IL-12) (Ağaç et al., 2018; **Figure 2**). This evidence may help clarify the relationship between peripheral and central inflammation and its modulation via the LC-NE system signaling.

### Glial contribution to neuroinflammatory progression

PD is characterized by the presence of Lewy bodies in the cytoplasm of dopaminergic neurons, formed by the aggregation of misfolded proteins, particularly α-syn (Huang et al., 2018; Tolö et al., 2018). The α-syn misfolded is associated with genetic mutations, especially in the SNCA (synuclein alpha) and LRRK2 (Leucine-rich repeat kinase 2) genes (Hu et al., 2018; Zafar et al., 2018; Liu et al., 2022). These mutations alter the peptide structure, modifying the characteristics and function of the protein, making it insoluble and, consequently, promoting the aggregation of oligomers and the production of reactive oxygen species (ROS) in the presynaptic region of dopaminergic neurons (Leão et al., 2015; Kang et al., 2018; Li et al., 2019).

In general, protein is believed to trigger several cytotoxic mechanisms, such as excessive production of ROS, mitochondrial dysfunction, dysregulation of intracellular calcium levels, among others, all of which culminate in a robust oxidative stress response and neuroinflammation, further exacerbated by neuronal death, particularly dopaminergic neurons (Trist et al., 2019; Yang and Zhou, 2019; Woodburn et al., 2021).

The progression of PD is associated with six neuropathological stages defined by α-syn deposition. The disease begins peripherally, affecting the olfactory bulb, vagus nerve, and enteric nervous system, then reaches the brainstem in stages 1–2, gradually ascends to the midbrain in stages 3–4, and finally spreads to the basal ganglia and cortical regions in stages 5–6 (Braak et al., 2003; Hawkes et al., 2007). These neuropathological stages are closely related to changes in the peripheral inflammatory profile, which may affect BBB permeability and, in turn, exacerbate central inflammatory responses to neuronal damage through disease progression (Yao et al., 2025).

In the early stages, peripheral α-syn aggregates can trigger an immune response by stimulating microglia and astrocytes through Toll-like receptors and inflammasomes (Isik et al., 2023). The sustained immune response by protein accumulation promotes the release of pro-inflammatory cytokines and ROS, thereby establishing a neurotoxic environment (Isik et al., 2023; Jurcau et al., 2023). In the intermediate stage, microglial activation becomes more pronounced, leading to increased production of pro-inflammatory factors prior to dopaminergic neuronal loss, which ultimately contributes to neurodegeneration (Jurcau et al., 2023). In the advanced stages of the disease, widespread dissemination of pathology to multiple cortical regions is associated with the infiltration of peripheral immune cells, further amplifying the inflammatory response and accelerating PD progression (Yao et al., 2025).

Faced with neuronal damage, glial cells adopt a pro-inflammatory phenotype, which is associated with morphological changes. Astrocytes shift from a star-shaped to a rounded morphology, increasing the release of pro-inflammatory factors such as IL-1β, TNF-α, IL-6, and nitric oxide. This promotes the weakening of the BBB, allowing infiltration of peripheral immune cells and accelerating cell death in neurodegenerative diseases (Kwon and Koh, 2020; Isik et al., 2023; Inchiosa, 2024; **Figure 1**). Protein aggregation itself can activate microglia and initiate the inflammatory cascade that leads to neurodegeneration (Badanjak et al., 2021).

Neuroinflammation is a key mediator of the processes involved in neurodegeneration. Studies indicate that central pro-inflammatory factors are become more prominent in the advanced stages of the disease, marked by increase activation of glial cells and infiltration of lymphocytes in patients with PD (Mcgeer et al., 1987; Smajić et al., 2022). Moreover, changes in the profile of peripheral immune markers and cells may contribute to the progression of PD (Brochard et al., 2009; Reale et al., 2009; Grozdanov et al., 2014; Hirsch and Standaert, 2021; Tansey et al., 2022; **Figure 1**). This perspective supports the hypothesis that dysfunction of the LC-NE system during aging or in the early stages of PD can promote immune system dysregulation and exacerbate neuroinflammation and neurodegeneration (Braak et al., 2003; Hawkes et al., 2007; Andersen et al., 2025).

Additionally, the overexpression of the glia maturation factor, which is involved in the growth and differentiation of glial cells and neurons, as well as in the inflammatory response in the CNS, activates mitogen-activation protein kinase and NF-κB pathways. This promotes glial activation, modulates glial cell phenotype, and influences the expression of pro-inflammatory cytokines, thereby contributing to neurodegeneration (Khan et al., 2014). The glia maturation factor also mediates apoptosis and regulates the expression of superoxide dismutase, granulocyte-macrophage colony-stimulating factor, and neurotrophins (Fan et al., 2018). Conversely, inhibition of nicotinamide adenine dinucleotide phosphate (NADPH) oxidase suppresses microglial activation, reduces the expression of pro-inflammatory factors, and prevents noradrenergic neurodegeneration in the LC (Hou et al., 2017), suggesting a strong link between neuroinflammation and noradrenergic signaling (**Figure 2**).

Although β-ARs are well recognized for their role in modulating neuroinflammation in neurodegenerative diseases, α-ARs are also involved in the regulation of neuroprotective mechanisms. In general, the α1-AR plays an important role in neural memory and plasticity (Perez, 2021). Their inhibition has been shown to reduce the production of neuroinflammatory factors through suppression of the NF-κB/NLRP3 pathway (Hussain et al., 2023). Accordingly, α1-AR stimulation is associated with an enhanced pro-inflammatory response in neurodegenerative diseases (Li et al., 2025). Moreover, selective antagonism of these receptors increases cellular ATP levels via phosphoglycerate kinase 1, thereby protecting dopaminergic neurons in PD (Cai et al., 2019; Weber et al., 2023; Lamichhane et al., 2024).

In addition to the α1-AR, α2-AR antagonists attenuate PD symptoms, particularly in combination with L-Dopa, by increasing monoamine availability in the synaptic cleft and reducing microglial activation as well as pro-inflammatory factors production (Henry et al., 1999; Yssel et al., 2018). Conversely, stimulation of these receptors with dexmedetomidine inhibits NF-κB1, regulating pro-inflammatory cytokine production and protecting dopaminergic neurons (Zhang et al., 2021). Although the α-ARs act directly or indirectly in modulating neuroinflammatory mechanisms, their role in DP progression remains poorly understood.

## β-Adrenergic Signaling in Neurodegeneration and Neuroprotection

### Clinical and experimental evidence linking β-adrenergic antagonism to neurodegenerative risk

The β-adrenoceptors antagonists are widely used to treat cardiovascular disease and hypertension but are also prescribed to alleviate certain anxiety symptoms (Morganroth et al., 1985; Stapleton, 1997; Archer et al., 2025). Recently, however, several studies have linked the chronic use of these drugs to an increased risk of neurodegenerative disorders, proposing that β-AR antagonism may amplify systemic inflammatory responses and thereby exacerbate neuroinflammation and neurodegeneration in the CNS (Oei et al., 2010; Evans et al., 2020, 2024; Hutten et al., 2022). In contrast, β-AR agonists appear to counteract these deleterious effects, promoting neuroprotection (Mittal et al., 2017; Gronich et al., 2018; Cepeda et al., 2019; Hopfner et al., 2019, 2020; Germay et al., 2020; O’Neill et al., 2020; Torrente et al., 2023; Tuominen et al., 2023; de Gois et al., 2025; Williams-Gray et al., 2025).

Additionally, recent studies have suggested that the increased risk of PD and other neurodegenerative diseases may be associated with the use of non-selective β-blockers (β1 and β2), particularly propranolol, but not with selective β-blockers (Mittal et al., 2017; Gronich et al., 2018; Cepeda et al., 2019; Singh et al., 2021; Hutten et al., 2022; Feng et al., 2023; Eijsvogel et al., 2024). In contrast, other studies attribute this association between the β-blockers and PD to reverse casualty (Searles et al., 2018; Germay et al., 2020; Giorgianni et al., 2020; Hopfner et al., 2020; Hopfner and Deuschl, 2021; Nguyen et al., 2025; Szmigiel et al., 2025; Williams-Gray et al., 2025).

During the prodromal phase of PD, patients may exhibit autonomic dysfunctions, such as cardiovascular abnormalities (Ariza et al., 2015; Palma, 2018; Menezes-Rodrigues et al., 2023) and tremors not yet associated with the disease, which are commonly treated with non-selective β-blockers such as propranolol (Searles et al., 2018). However, it remains unclear whether the chronic use of β-blockers may accelerate disease progression.

Despite the correlational nature of the findings, a study suggests that propranolol may exert cytotoxic effects by increasing mitochondrial toxicity and ROS production, as well as by exacerbating the pro-inflammatory response (Evans et al., 2024). It has also been shown to enhance α-syn accumulation (Mittal et al., 2017) and α-syn-mediated neurodegeneration (Torrente et al., 2023). Additionally, propranolol may inhibit acetylcholinesterase and ATPase activity (Seydi et al., 2020), and impair learning and memory (Goodman et al., 2021), all of which are factors that may contribute to neurodegeneration.

Although epidemiological studies have indicated an association between propranolol use and an increased risk of PD, and preclinical studies have reported cytotoxic effects, propranolol also functions as a membrane stabilizer by blocking ion channels (Black et al., 1964; Stapleton, 1997; Al-Majed et al., 2017), and is used in the treatment of essential tremor as well as L-Dopa-induced late-onset dyskinesia (Crosby et al., 2003; McKinley et al., 2020; Shi et al., 2020; Hopfner and Deuschl, 2021), as this β-blocker reduces the intensity and amplitude of contralateral motor cortex tremors, both in the presence and absence of stress (Heide et al., 2024).

We recently discovered that this β-blocker can promote neuroprotection in a rodent model of parkinsonism (de Gois, et al., 2025). Although the neuroprotective mechanisms remain unclear, studies suggest that propranolol may promote an anti-inflammatory phenotype in macrophages by upregulating the expression of CD163, CD206, and IL-10 (Abdin et al., 2014), and by inhibiting TNF-γ signaling, suppressing IL-1β synthesis in human monocytes though the blockade of diacylglycerol formation via phospholipase D (Luong and Nguyễn, 2013), which are mechanisms involved in modulation of immune and inflammatory response.

Additionally, propranolol-induced macrophage activation may enhance the expression antioxidant proteins via nuclear factor erythroid 2-related factor 2 (Nrf2), a transcription factor involved in regulating cytoprotective responses (Maccari et al., 2024). Given that elevated oxidative stress is a key contributor to neurodegenerative diseases, particularly PD (Maccari et al., 2024; Mohammed et al., 2024), Nrf2-mediated antioxidant production may represent an important neuroprotective mechanism stimulated by propranolol or other non-selective β-blocker. In this context, inhibition of β-AR signaling in glial cells may reduce ROS production and neuronal damage through extracellular signal-regulated kinases (ERK)-dependent pathway (Qian et al., 2009; Sugama et al., 2019). However, the diverse mechanisms of propranolol complicate the understanding of its overall impact on neurodegeneration.

### β2-Adrenergic agonists as emerging therapeutics for neuroinflammation and neuronal protection

Despite the controversial relationship between β-AR antagonists and neurotoxic effects in neurodegenerative diseases, particularly in PD, a growing body of evidence supports a strong association between β2-AR stimulation and neuroprotective mechanisms in the PD and other neurodegenerative disease (Mittal et al., 2017; Gronich et al., 2018; Cepeda et al., 2019; Germay et al., 2020; O’Neill et al., 2020; Singh, 2020; Hutten et al., 2022; Torrente et al., 2023; de Gois et al., 2025; Nguyen et al., 2025). These Neuroprotective mechanisms include the downregulation of α-syn expression (Mittal et al., 2017; de Gois et al., 2025), as well as the attenuation of neuroinflammatory responses and oxidative stress mediated by β2-AR activation (Evans et al., 2020; O’Neill et al., 2020; Torrente et al., 2023).

A clinical study has shown that the β2-AR agonist clenbuterol increases blood flow in various brain regions, such as the hippocampus, amygdala, thalamus, and precentral gyrus, in both healthy individuals and patients with mild cognitive impairment or PD, suggesting enhanced neuronal activity and cognitive improvement in these areas (Lodeweyckx et al., 2024). The increase in cerebral blood flow may be related to the presence of β2-ARs in astrocytes (Paukert et al., 2014; Magistrelli and Comi, 2020), as the modulation of these receptors by NE or exogenous agonists promotes an increase in calcium ion (Ca^2+^) signaling within astrocytes (Paukert et al., 2014), leading to arteriolar dilation and functional hyperemia (Institoris et al., 2022). Moreover, the concomitant administration of a β2-AR agonist and L-Dopa has been shown to facilitate the transport of L-Dopa across the BBB, thereby enhancing its therapeutic efficacy (Takao et al., 1992; Uc et al., 2002).

Another recent clinical study also showed that short-acting β2-AR agonists, such as salbutamol, and long-acting agonists, such as clenbuterol, improve cognitive functions in patients with PD, with a more pronounced effect observed for clenbuterol (Eijsvogel et al., 2024). However, the small sample size and short treatment duration in these studies highlight the need for more rigorous, controlled clinical studies to better evaluate the promising therapeutic effects of β2-AR agonists in neurodegenerative diseases (Oei et al., 2010; Mittal et al., 2017; Zong et al., 2019; Evans et al., 2020, 2024; O’Neill et al., 2020; Khidr et al., 2023; Torrente et al., 2023; de Gois et al., 2025).

Studies using experimental models have shown that LC-NE system degeneration accelerates dopaminergic neuron loss, whereas elevated NE levels or β2-AR stimulation can protect dopaminergic neurons by modulating neuroinflammatory pathways. This includes the inhibition of the pro-inflammatory microglial phenotype and the suppression of migration of immune cells into the CNS (Yssel et al., 2018; Kreiner et al., 2019; O’Neill et al., 2020; Zhu et al., 2022; Torrente et al., 2023). Ryan et al. (2013) demonstrated that a single systemic injection of clenbuterol or formoterol was sufficient to suppress the activity of pro-inflammatory NF-κB induced by intracerebroventricular injection of lipopolysaccharide.

Additionally, we recently demonstrated that daily administration of salbutamol for eight days, starting after the onset of motor deficits in a progressive reserpine-induced parkinsonism model, improved motor performance, protected dopaminergic neurons, and prevented the upregulation of α-syn expression (de Gois et al., 2025). However, this same study also showed that β-AR blockade with propranolol exerted neuroprotective effects.

Despite strong evidence supporting the neuroprotective role of β2-AR stimulation in PD, it appears that the effects modulation these receptors may vary depending on the types of experimental model, disease stage, severity, and the underlying pathophysiological mechanisms involved in the progression (**Additional Table 1**). Therefore, further studies are needed to determine the most effective strategies and optimal timing of β-AR modulation throughout the course of neurodegeneration.

**Additional Table 1:** Summary of preclinical, clinical and epidemiological studies on β-adrenergic modulation in Parkinson’s disease

Study type	Intervention	Model/Population	Main findings	Reference
**Preclinical**	**β2-AR agonists:** Salbutamol (10 μmol/kg, acute) Clenbuterol (0.5 mg/kg, acute) Isoproterenol (non-selective β-AR agonist, 10 μmol/kg and 2.5 mg/Kg, acute) Dobutamine (β1-AR agonist, 50 μmol/kg, acute) **Non-selective β-AR antagonist:** Propranolol (50 μmol/kg, acute) Nadolol (10 mg/kg, acute)	Wistar rats	β2-AR agonists (isoproterenol and salbutamol) regulate aromatic amino acid transport across the BBB; effect blocked by propranolol.	Takao et al., 1992; Uc et al., 2002
**Preclinical**	**β2-AR agonist:** Salmeterol (1-10 μg/kg doses, chronic)	LPS and MPTP mouse model of PD	Salmeterol reduced microglial activation, pro-inflammatory cytokines and preserved dopaminergic neurons.	Qian et al., 2011
**Preclinical**	**β2-AR agonists:** Clenbuterol (0.5 mg/kg, acute) Formoterol (0.5 mg/kg, acute) **Antagonist β-AR:** Propranolol (non-selective, 10 mg/kg, acute) Metoprolol (selective β1-AR, 10 mg/kg, acute) ICI-118,551 (selective β2-AR, 10 mg/kg, acute)	LPS rat model	Clenbuterol and formoterol suppressed NF-κB-driven inflammation; effect blocked by propranolol or ICI 118.551, but not by metoprolol.	Ryan et al., 2013
**Preclinical**	**β2-AR agonist:** Clenbuterol (10 mg/kg, chronic)	MPTP mouse model of PD	Clenbuterol promoted dopaminergic neuroprotection in MPTP model.	Mittal et al., 2017
**Preclinical**	**β2-AR agonists:** Clenbuterol (100 μg/kg, acute) Formoterol (100 μg/kg, acute) Seven days of administration for all drugs.	LPS rat model of PD	Clenbuterol and formoterol reduce neuroinflammation and protected dopaminergic neurons.	O’Neill et al., 2020
**Preclinical**	**Non-selective antagonist β-AR:** Propranolol (5 mg/kg and 20 mg/kg, chronic)	*Slc6a3^DTR /+^* mouse model of PD	Propranolol reduced L-Dopa-induced dyskinesia via β-AR modulation of striatal interneurons.	Shi et al., 2020
**Preclinical**	**β2-AR agonist:** Clenbuterol (10 mg/kg, chronic) **β1-AR agonist:** Xamoterol (3 mg/kg, chronic) **Non-selective β-AR antagonist:** Propranolol (10 mg/kg, chronic)	hα-Syn mouse model of PD	β2-AR stimulation (clenbuterol) reduce α-syn, neuroinflammation, and loss dopaminergic, whereas β1-AR agonist (xamoterol) increased inflammation, BBB permeability, and neurodegeneration.	Torrente et al., 2023
**Preclinical**	**β2-AR agonists:** Levalbuterol (4 μg/mL, acute) Albuterol (8 μg/mL, acute) Arformoterol (0.008 μg/mL, acute) Five days of administration for all drugs.	Female immunodeficient NSG-SGM3 mouse	Levalbuterol, arformoterol suppressed central and peripheral inflammation.	Inchiosa, et al., 2024
**Preclinical**	**β2-AR agonist:** Salbutamol (5 mg/kg, acute) **Non-selective antagonist β-AR:** Propranolol (20 mg/kg, acute) Eight days of administration for all drugs.	Reserpine rat model of DP	Salbutamol and propranolol prevent TH/DβH loss and a-syn increase, promoting neuroprotection.	de Gois et al., 2025
**Clinical**	**Non-selective antagonist β-AR:** Propranolol (40 mg)	Cross-over, double-blind intervention study (patients with PD - men and women)	Propranolol reduced Parkinsonian tremor, including stress conditions.	Van der Heide et al., 2024
**Epidemiological**	**β2-AR agonist:** Salbutamol **Non-selective β-AR antagonists:** Propranolol Carvedilol **Selective β1-AR antagonist:** Metoprolol	Population-based case-control study of US Medicare beneficiaries (period 2004-2009)	Salbutamol not associated with PD risk; propranolol increased PD risk, but effect disappeared after adjusting for tremor. Carvedilol and metoprolol reduce risk.	Searles et al., 2018
**Epidemiological**	**β2-AR agonist:** Salbutamol **Non-selective antagonist β-AR:** Propranolol	Norwegian population cohort study (period 2005-2014)	Salbutamol associated with reduced PD risk; propranolol with increased risk (likely reverse causality).	Mittal et al., 2017
**Epidemiological**	**Short-acting β2-AR agonists:** Salbutamol Terbutaline **Long-acting β2-AR agonists:** Salmeterol Formoterol **Ultra-long-acting β2-AR agonists:** Vilanterol Indacaterol Olodaterol **Non-selective β-AR antagonists:** Propranolol Carvedilol Sotalol Labetolol Pindolol **Selective β1-AR antagoists:** Atenolol Metropolol Bisoprolol	Case-Control Study in a Cohort Israeli (period 2004-2017)	Non-selective β-antagonists increased PD risk; selective β1-blockers did not. β2-agonists reduced PD risk; associations persisted even 5 years before diagnosis.	Gronich et al., 2018
**Epidemiological**	**β2-AR agonist:** Albuterol **Non-selective β-AR antagonist:** Propranolol	Self-controlled cohort study - US databases (period 2000-2018)	Albuterol reduced PD risk; propranolol increased PD risk.	Cepeda et al., 2019
**Epidemiological**	**Short-acting β2-AR agonists:** Salbutamol Terbutaline **Long-acting β2-AR agonists:** Salmeterol Formoterol **Non-selective β-AR antagonists:** Propranolol Sotalol **Selective β1-AR antagoists:** Atenolol Metropolol Bisoprolol	Case-control study - Danish population (period 2000-2012)	β2-AR agonists reduced PD risk; β2-AR antagonist increased risk. Protective effect partly mediated by smoking; antagonist effect suggested indicates reverse causality.	Hopfner et al., 2019
**Epidemiological**	**β2-AR agonist:** Salbutamol **Non-selective antagonist β-AR:** Propranolol	Case-control study - French Insurance System (period 2008-2017)	Propranolol increased PD risk; β2-AR agonists protective, but only in non-diabetic patients.	de Germay et al., 2020
**Epidemiological**	Short- and long-acting selective β2-AR agonists and Non-detective β2-AR antagonists	Cohort study, case-control - UK Clinical Practice Research Datalink (period 1995-2016)	β2-agonists reduced PD incidence (short-term use). β-blockers increased PD risk, especially short-term, supporting reverse causality.	Giorgianni et al., 2020
**Epidemiological**	Non-selective β-AR blockers Selective β1-AR blockers (SBBs)	Disproportionality analysis; UK Biobank cohort analysis (period 2006-2010); Mendelian randomization (MR) analysis	Non-selective β-AR blockers linked to PD; selective β1-AR blockers not. Higher β2-AR expression associated with reduced PD risk (MR analysis).	Feng et al., 2023
**Epidemiological**	Short-acting β2-AR agonists (SABAs) Long-acting β2-AR agonists (LABAs) Ultra-long-acting β2-AR agonists (ultra-LABAs)	Norwegian population cohort study (period 2005-2019)	Short-, long-, and Ultra-long-acting β2-AR agonist all inversely associated with PD risk, Strongest with ultra-LABA.	Touminen et al., 2023
**Epidemiological**	Short-acting β2-AR agonists (SABAs) Long-acting β2-AR agonists (LABAs) Ultra-long-acting β2-AR agonists (ultra-LABAs) Non-selective β-AR antagonists Propranolol and other Selective β1-AR antagonists	Case-control study, E3N cohort - French women (period 1990-2018)	LABAs and ultra-LABAs (but not SABAs) associated with reduced PD incidence. β-blockers linked to PD risk without lag, especially propranolol, suggesting reverse causality.	Nguyen et al., 2025

α-syn: α-Synuclein; β-AR: β-adrenoceptor, β1-AR: β1-adrenoceptor; β2-AR: β2-adrenoceptor; BBB: Blood-brain barrier; DβH: dopamine β-hydroxylase; hα-syn: human α-synuclein; ICI-118,551 : selective β2-adrenergic receptor antagonist; LABAs: Long-acting β2-AR agonists; L-Dopa: L-3,4-dihydroxyphenylalanine; LPS: lipopolysaccharide; MPTP: 1-methyl-4-phenyl-1,2,3,6-tetrahydropyridine; MR: Mendelian randomization; NF-κB: nuclear factor kappa B; NSG-SGM3: NOD scid gamma mice expressing human stem cell factor, granulocyte-macrophage colony-stimulating factor, and interleukin-3; PD: Parkinson’s disease; SABAs: short-acting β2-adrenergic receptor agonists; SBBs: selective β-AR blockers; *Slc6a3^DTR/+^*: solute carrier family 6 member 3 - diphtheria toxin receptor; TH: tyrosine hydroxylase; ultra-LABAs: ultra-long-acting β2-AR agonists.

### Intracellular signaling pathways underlying β2-adrenoceptors-mediated neuroprotection

Growing evidence supports the neuroprotective role of β2-AR agonist in neurodegenerative diseases, particularly in PD. However, the underlying neuroprotective mechanisms associated with β2-AR activation remain unclear. To date, it is known that these receptors can modulate the inflammatory phenotype of microglial cells (Hristovska and Pascual, 2016), astrocytes (Magistrelli and Comi, 2020; Chen et al., 2023; Verkhratsky et al., 2023), and immune system cells (Magistrelli and Comi, 2020). Additionally, β2-AR activation may enhance neurogenesis, dendritic branching, and synaptic density in the CNS (Chai et al., 2016). Therefore, a deeper understanding of the intracellular signaling mechanisms triggered by β2-AR is essential to guide the development of more effective therapeutic strategies.

The β2-ARs are glycoprotein family members of the seven-transmembrane G protein-coupled receptor superfamily. In general, canonical β2-AR stimulation via Gs protein coupling activates adenylate cyclase, increasing intracellular levels of cyclic adenosine monophosphate (cAMP), which in turn activates protein kinase A (PKA), leading to the phosphorylation of various regulatory protein targets (Johnson, 2006). However, β2-ARs can also signal through non-canonical pathway, including the recruitment of β-arrestins (Drake et al., 2008). Therefore, the diversity of signaling pathways mediated by β2-ARs, along with variations in agonist concentration and subtype, may complicate the understanding of the neuroprotective mechanisms associated with these receptors.

Neuroprotection mediated by β2-AR stimulation appears to be associated with the inhibition of transcription factors that regulate inflammatory response, such as NF-κB. Ryan et al. (2013) showed that clenbuterol or formoterol suppresses NF-κB subunit P65, increases the expression of the inhibitor of kappa B alpha (IκBα) gene and protein, and inhibits IκBα phosphorylation and degradation. This leads to the inhibition of NF-κB subunit translocation to the nucleus and consequently reduces the expression of pro-inflammatory cytokines and intercellular adhesion molecule-1. Although not fully understood, the authors suggest that the increase in IκBα synthesis is associated with the classic β2-AR signaling pathway, involving the activation of PKA via elevated cAMP levels (Ryan et al., 2013; **Figure 2**).

Interestingly, β2-AR agonists can activate mitogen-activated protein kinase (MAPK)-mediated signaling cascades and promote pro-inflammatory phenotypes, either in a cAMP-dependent or -independent manner, or non-canonical β-arrestin recruitment. In addition to the classic Gs protein activation and cAMP increase via the canonical pathway, β-AR can also signal through non-canonical pathways involving β-arrestins. β-arrestins are adaptor proteins that bind to phosphorylated receptors, promoting their desensitization and endocytosis, but they also participate in signaling cascades by recruiting kinases and other molecules that modulate MAPK, PI3K/AKT (protein kinase B) pathways, ultimately regulating NF-κB-mediated inflammation independently of G-protein (Gao et al., 2004; Fang et al., 2021).

Many β2-A agonists activate β-arrestin-mediated signaling, whereas β1-AR can trigger both β-arrestin1 and 2 (Gu et al., 2015). However, in the context of neuroinflammation, β2-ARs are considered more relevant, as β2-A agonists reduce microglial activation and the release of pro-inflammatory factors (Quian et al., 2011). Moreover, recent findings indicate that β-arrestin2 depletion in a DP model exacerbates microglia-mediated neuroinflammation, while β-arrestin1 depletion protects against dopaminergic neurodegeneration (Fang et al., 2021), highlighting opposite effects in PD. These findings reinforce the contrasting role of β2-AR and β1-AR stimulation in neuroinflammation and dopaminergic neurodegeneration (Torrente et al., 2023), further supporting the neuroprotective role of β2-AR modulation.

The β2-AR agonists have been shown to increase in the expression of pro-inflammatory factors via MAPK activation through enhanced phosphorylation of ERK and NADPH oxidase, mediated by cAMP (Hung et al., 2008; McNamee et al., 2010). However, other studies have reported the activation of this pathway independently of cAMP, PKA, and β-arrestin (Tan et al., 2007; Qian et al., 2009), although β-arrestins are known to activate MAPK signaling in various cell types (Luttrell and Lefkowitz, 2002). Conversely, the anti-inflammatory effect has been associated with inhibition of the p65 subunit of NF-κB and the phosphorylation of MAPK members, including ERK, p38, and JNK, through β-arrestin recruitment in a cAMP-independent manner (Qian et al., 2011; **Figure 2**). Moreover, the recruitment of β-arrestins in complex with kinases has been shown to sustain mitogenic activation and regulation of pro-inflammatory factors (Gu et al., 2015). Additionally, β2-AR stimulation may promote β-arrestin2 binding to the NF-κB inhibitor IκBα, preventing its phosphorylation and degradation, thereby blocking NF-κB nuclear translocation and decreasing pro-inflammatory gene expression (Gao et al., 2004). Thus, modulation of inflammation via β2-AR depends on the cell type, the nature of the injury, and the experimental model of neurodegeneration employed.

In contrast, some β-blockers have been reported to function as biased agonists, suppressing canonical Gs signaling while recruiting β-arrestin2 to activate the G protein-independent ERK pathway (Gu et al., 2015; Ibrahim et al., 2021). However, the effects of β-blockers on neuroinflammation may also be partially attributable to actions beyond β-RA blockade, including membrane stabilization, ion channel modulation, and antioxidant properties, which ultimately reduce the inflammatory response (Black et al., 1964; Stapleton, 1997; Al-Majed et al., 2017). This mechanism may underline potential anti-inflammatory effects mediated by β-arrestin. Nevertheless, evidence supporting these effects in PD remains scarce.

Analyses using an experimental model of parkinsonism have shown that formoterol increases β2-AR expression and stimulates the canonical signaling cascade, leading to elevated levels of cAMP, activation of PKA, phosphorylation of cAMP response element-binding protein (CREB), and synthesis of the brain-derived neurotrophic factor. The elevation of brain-derived neurotrophic factor levels is proportional to stimulation of the PI3K/AKT/CREB signaling pathway, which inhibits NF-κB p65 activity and α-syn expression, thereby promoting neuroprotection (Jurič et al., 2008; Zhu et al., 2019; Khidr et al., 2023; **Figure 2**). In addition, clenbuterol and salbutamol also promote neuroprotection in experimental models of parkinsonism by downregulating α-syn gene expression and reducing soluble α-syn immunoreactivity (Mittal et al., 2017; de Gois et al., 2025), which is the key pathological protein in PD.

## Concluding Remarks and Future Directions

The β2-ARs appear to modulate several neuroprotective mechanisms that support neuronal survival, including both peripheral and central inflammation, as well as protein aggregation pathology. However, the stage of neurodegeneration and the types of neuronal injury seem to influence the recruitment of specific signaling pathways that may either protect neuronal or exacerbate neuronal damage. Therefore, further mechanistic analyses are required to evaluate the effects of short- and long-action β2-AR agonists, dosing concentrations, stages of neurodegeneration, and interactions with other neurotransmitter systems to clarify which intracellular signaling cascades are activated and how they contribute to neuroprotection.

## Additional file:

***Additional Table 1:*** *Summary of preclinical, clinical, and epidemiological studies on* β*-adrenergic modulation in Parkinson’s disease.*

## Data Availability

*All relevant data are within the paper and its Additional files.*
